# AsianVehicle: An image dataset of traditional Asian vehicles

**DOI:** 10.1016/j.dib.2026.112709

**Published:** 2026-03-20

**Authors:** Md. Darun Nayeem, Md. Sadikujjaman, Rehnuma Tabassum, Anika Ivnath, Md. Masudul Islam

**Affiliations:** a Bangladesh University of Business and Technology, Dhaka, Bangladesh; b Daffodil International University, Daffodil Smart City, Dhaka, Bangladesh

**Keywords:** Traditional asian vehicles, Transport dataset, Vehicle image classification, Cultural heritage preservation

## Abstract

The AsianVehicle dataset introduces a comprehensive image collection of four traditional Bangladeshi vehicle types—Auto Rickshaw, Rickshaw, Rickshaw Van, and Leguna—captured to support research in computer vision and cultural informatics. These vehicles, integral to South and Southeast Asian transport systems, represent unique visual and structural characteristics seldom documented in existing global datasets. A total of 4000 RGB images were collected across various urban and semi-urban areas of Mirpur, Dhaka, using smartphone cameras under natural daylight conditions to preserve authentic colors, textures, and environmental diversity. The dataset encompasses variations in viewing angles, backgrounds, and illumination, reflecting real-world scenarios where such vehicles operate. All images are provided in properly processed form, enabling users to apply customized preprocessing, and labeling strategies according to their research needs. Beyond supporting machine learning tasks such as vehicle classification, segmentation, or detection, this dataset contributes to the digital preservation of traditional Asian transport designs that are gradually disappearing due to modernization. Its open accessibility facilitates comparative studies on model generalization, cross-domain adaptation, and low-resource visual recognition. By bridging cultural representation and artificial intelligence research, AsianVehicle offers a valuable foundation for both technical innovation and the preservation of regional identity within data-driven applications.

Specifications Table

**Table:** 

Subject	Computer Sciences
Specific subject area	Computer Vision, Image Recognition, and Cultural Informatics, Digital Preservation of Traditional Vehicles
Type of data	*Image*
Data collection	*The dataset comprises four traditional vehicle types commonly found in Bangladesh—Rickshaw, Rickshaw Van, Leguna, and Auto Rickshaw (CNG)—collected from diverse urban and semi-urban areas of Mirpur, Dhaka. Images were captured in local markets and surrounding neighborhoods using Redmi Note 9 and Vivo Y11 smartphones under natural daylight conditions at ambient temperatures of 25* °*C to 28* °*C to preserve authentic color and texture. Each vehicle was photographed from multiple angles and distances to reflect real-world variability in appearance, lighting, and background. The dataset contains 4000 RGB images that highlight the distinctive visual features of each vehicle type.*
Data source location	*Latitude 23.8103° N, Longitude 90.4125° E*
Data accessibility	Repository name: **Mendeley Data** Data identification number: 10.17632/4ccfy8hdkw.1 Direct URL to data: https://data.mendeley.com/datasets/4ccfy8hdkw/2/ To access the dataset, visit the provided Mendeley Data preview link. On that page, you will find the “Download” button. Accept the terms if prompted, and download the ZIP archive. The archive 4000 JPG files sorted into four class folders. These files can be directly imported into machine‑learning frameworks. No request for additional access is required securely.
Related research article	*None*

## 1 Value of the Data

• The dataset captures authentic transport diversity from South Asia—a region rarely represented in global vehicle image repositories dominated by Western automobile types. It enables fair benchmarking and cross-cultural evaluation of computer vision models, supporting research on bias mitigation and geographical generalization.
• Naturally varied lighting, cluttered backgrounds, and mixed viewpoints mirror genuine urban conditions typical of developing cities. These uncontrolled environments make the dataset ideal for robustness testing, transfer learning, and domain adaptation experiments under real-world constraints.
• Each image reflects distinctive craftsmanship and the ongoing transition from human-powered to semi-motorized mobility in South Asia. Beyond technical value, the dataset contributes to the digital preservation of region-specific transport heritage now fading from public roads.
• The class-balanced, and non-Western imagery provides strong substrates for active learning, self-supervised pretraining, and few-shot or open-set recognition. It also offers a realistic testbed for auditing fairness, visual drift, and model adaptation in diverse cultural contexts.
• For benchmarking purposes, researchers may apply a standard 80/10/10 or 70/15/15 train/validation/test split across the four classes. Baseline experiments using standard architectures such as ResNet-50, EfficientNet-B0, or Vision Transformer models can provide useful reference performance for classification tasks.

## 2 Background

Rapid progress in computer vision and deep learning has led to extensive development of vehicle recognition systems [1,2]; however, most available datasets are concentrated on Western automobiles [3] and organized traffic environments [4,5]. This imbalance limits the adaptability of AI models to regional transportation systems in South and Southeast Asia, where local and hybrid vehicles dominate the streets. To address this gap, the *AsianVehicle* dataset was compiled to document four widely used Bangladeshi vehicles—Auto Rickshaw, Rickshaw, Rickshaw Van, and Leguna—that represent distinct structures, materials, and operational contexts. The dataset was created under a methodological framework emphasizing natural, in-situ image acquisition using smartphone cameras, ensuring authentic lighting, background, and perspective variation. It was designed to support the development and validation of machine learning methods that can handle diverse real-world imagery without controlled laboratory conditions. By offering a regionally grounded visual resource, this dataset provides researchers with a foundation for benchmarking, training, and comparing computer vision algorithms focused on localized transport systems and underrepresented cultural contexts.

## 3 Data Description

**Fig. 1:**
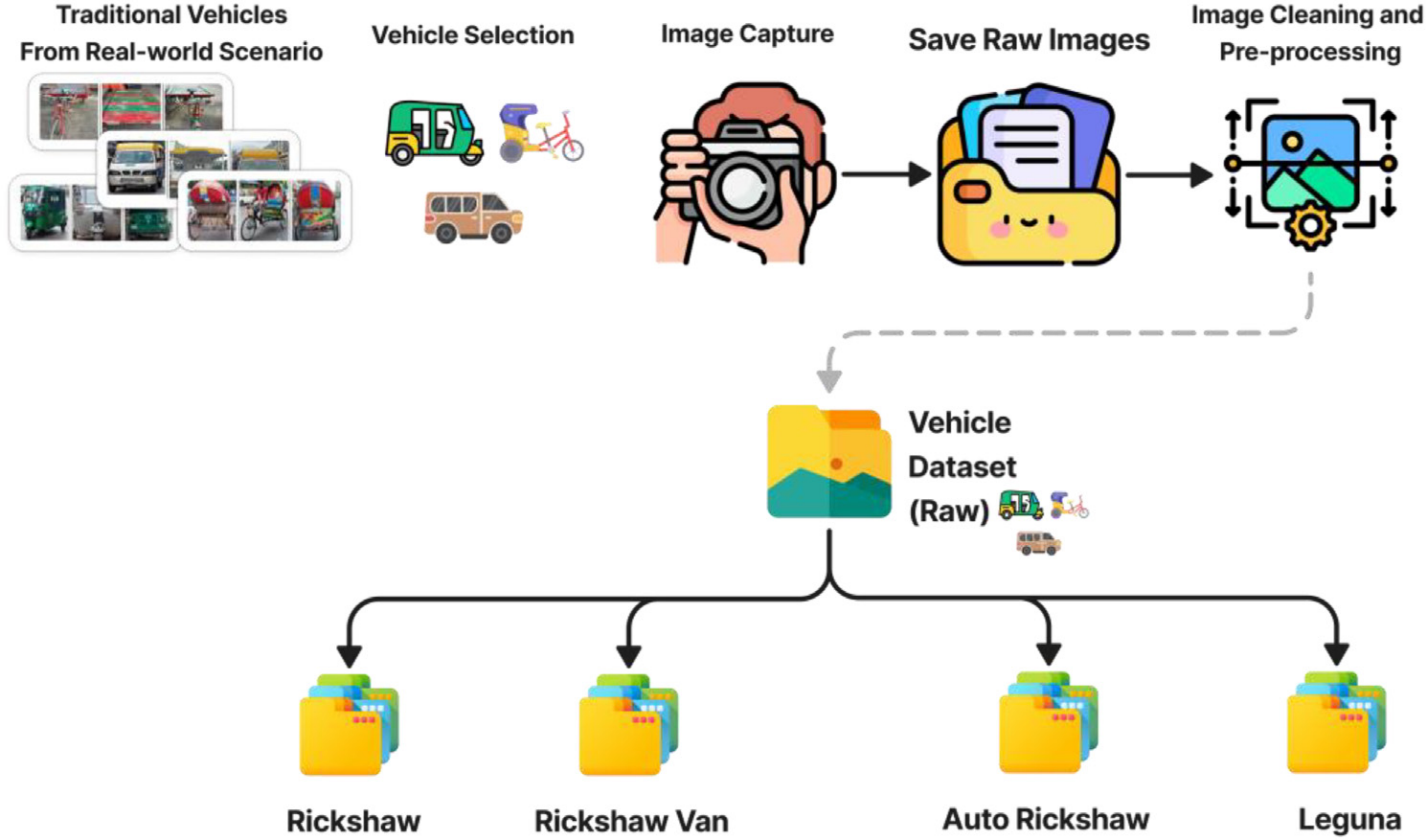
Dataset collection procedure and folder organization.

The AsianVehicle [6] dataset is a Mirpur, Dhaka–focused image collection organized into four main folders, each representing one vehicle class: Auto Rickshaw (CNG), Rickshaw, Rickshaw Van, and Leguna. Each folder contains 1000 unique RGB images captured under natural daylight conditions using smartphone cameras. All images are provided in their resized form to maintain color accuracy, texture, and environmental context. A consolidated CSV metadata file (filename, class label, resolution, and capture timestamp extracted from EXIF) is provided alongside the dataset to support reproducibility and downstream analysis. The process shown in Fig 1. Each image is stored in JPG format and named using a unique identifier. Images were captured from varying angles—front, side, and oblique—under ambient daylight conditions. All images containing human presence were carefully blurred or anonymized to protect individual privacy while maintaining the integrity of the vehicle data. The dataset is released under an open-access license (CC BY 4.0), allowing reuse with attribution. While segmentation examples using the Segment Anything Model (SAM) are presented for demonstration purposes, pixel-level annotation masks are not currently provided. Future releases aim to include standardized annotation formats (COCO-compatible labels) and expanded metadata to further support detection and segmentation research.

**Fig. 2:**
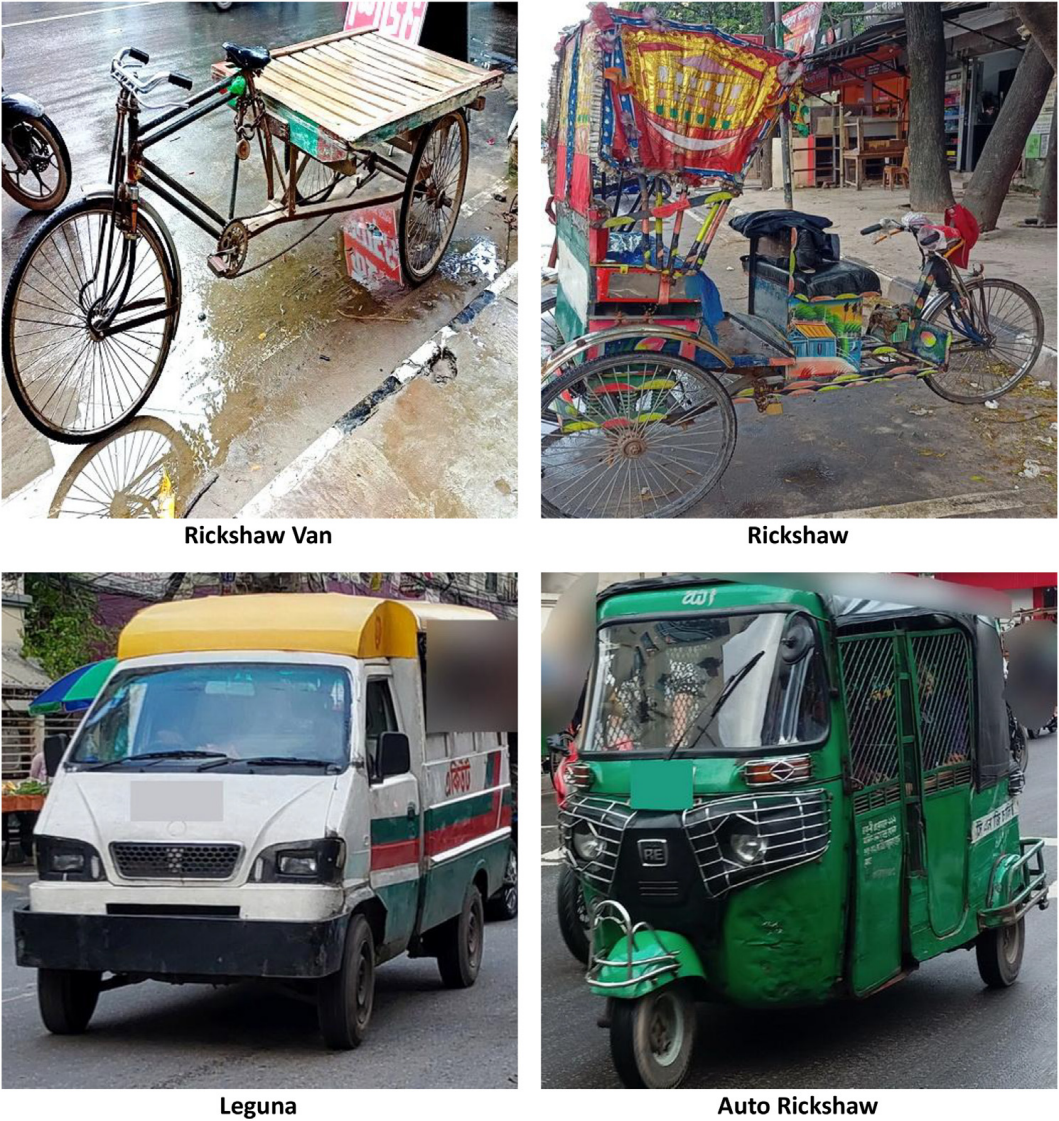
Sample images of AsianVehicle each class.

Table 1 summarizes our dataset descriptions, basic and visual features and sample images. In Table 2 presents the overall image meta-data information is described. Sample images from each class is represented in Fig 2.

**Table 1:** Dataset description and sample images.

Class & Example Images	Visual Features and Description
Rickshaw	✓ **Visual Characteristics:** Manually pulled tricycle with artistic wooden frame and decorative canopy. ✓ **Common Color & Material:** Brightly painted with floral and cultural motifs; metal and wood. ✓ **Distinguishing Features:** Human-powered, high artistic rear panels, exposed seating area, hand-painted body.
Auto Rickshaw (CNG)	✓ **Visual Characteristics:** Three-wheeled semi-motorized vehicle with a covered metal frame and open sides. ✓ **Common Color & Material:** Green, yellow, or mixed; metal body with vinyl covers. ✓ **Distinguishing Features:** Compact design, rear engine, registration plate at back, distinctive green shade typical of urban CNGs.
Leguna	✓ **Visual Characteristics:** Locally modified motorized carrier resembling a mini-van or shared taxi. ✓ **Common Color & Material:** Multicolor body with metal chassis and roof extensions. ✓ **Distinguishing Features:** Wider seating, customized front cabin, extended roof, often used for rural group transport.
Rickshaw Van	✓ **Visual Characteristics:** Larger tricycle designed for goods and passenger transport with flat open space. ✓ **Common Color & Material:** Metallic frame with wooden platform; less decorative. ✓ **Distinguishing Features:** Horizontal flatbed structure, rectangular rear, more utilitarian in appearance.

**Table 2:** Image and metadata information.

Parameter	Details
Total Images	4000
Per Class Image	1000
File Format	JPG (RGB)
Image Resolution Range	Resized to 512×512
Average File Size	0.14- 0.15 MB per image
Total Dataset Size	111 MB (ZIP Format)
Meta Data Information	Asianvehicle_metadata.csv
Image Source Devices	Redmi Note 9, Vivo Y11 (Smartphone Camera)
Capture Temperature Range	25 °C–28 °C
Capture Conditions	Natural daylight, outdoor markets and roadside settings

## 4 Experimental Design, Materials, and Methods

Data collection was conducted over approximately 10 non-consecutive days during March–April 2025, representing around 10 independent capture sessions distributed across multiple locations in Mirpur, Dhaka. Data collection followed a structured neighborhood coverage plan, focusing on several transport-dense zones including Mirpur-1, Mirpur-10, and Mirpur-11. Image capture sessions were distributed across late morning and afternoon periods (10:00 AM–4:00 PM) to represent common daylight operating conditions of these vehicles. Although geographically limited to Mirpur, the dataset captures diverse roadside environments such as markets, residential streets, and transport hubs. During these sessions, vehicles passing through public roads and markets were photographed opportunistically. Based on manual inspection during duplicate screening, the dataset is estimated to contain images of approximately 200–300 distinct vehicles, although multiple viewpoints of the same vehicle were intentionally retained to capture structural variability and realistic observational conditions. Class balance (1000 images per category) was achieved by continuing collection for underrepresented vehicle types until equal sample counts were obtained across all four classes. Images were captured using Redmi Note 9 and Vivo Y11 smartphones operating in default auto-camera mode, with autofocus enabled and automatic exposure control. No manual adjustment of ISO, shutter speed, white balance, or focal length was applied, and HDR was disabled. Native capture resolutions ranged from 960×720 to 768×1024 pixels. Vehicles were photographed from multiple viewpoints (front, side, rear, and oblique) and at approximate distances of 1.5–4 m to capture structural variability under real-world conditions. A structured post-collection workflow was applied: (i) initial quality screening, (ii) anonymization, (iii) labeling, (iv) duplicate detection, and (v) final dataset organization. Images exhibiting severe motion blur, excessive occlusion, or incomplete vehicle visibility were excluded during quality screening. De-identification was performed using Adobe Photoshop 2023 and GIMP 2.10. Visible human faces, exposed body parts, and vehicle license plates were blurred using Gaussian blur, while preserving vehicle structure and contextual background. Any region was considered “identifiable” if facial features or alphanumeric plate characters were visually discernible. No other image enhancement or augmentation was applied. Class labels were independently assigned by two authors based on distinctive structural characteristics of each vehicle type (e.g., enclosed motorized cabin and extended roof for Leguna; flat rear cargo platform for Rickshaw Van). Disagreements were resolved through joint review. A quality assurance pass was subsequently performed in which approximately 10% of samples were randomly rechecked for labeling consistency. Duplicate and near-duplicate images were initially screened through manual visual inspection across folders, followed by filename and resolution comparison. Additional checks were conducted by comparing visually similar samples captured in rapid succession to ensure that only distinct viewpoints were retained. Multiple images of the same vehicle were intentionally preserved to capture viewpoint variability; however, exact counts of unique vehicles were not recorded. All released images were resized to 512×512 pixels and organized into four class-specific folders containing 1000 images each. A consolidated CSV metadata file accompanies the dataset, mapping filename, class label, capture device, image resolution, and timestamp extracted from EXIF data to support reproducibility and downstream reuse.

**Fig. 3:**
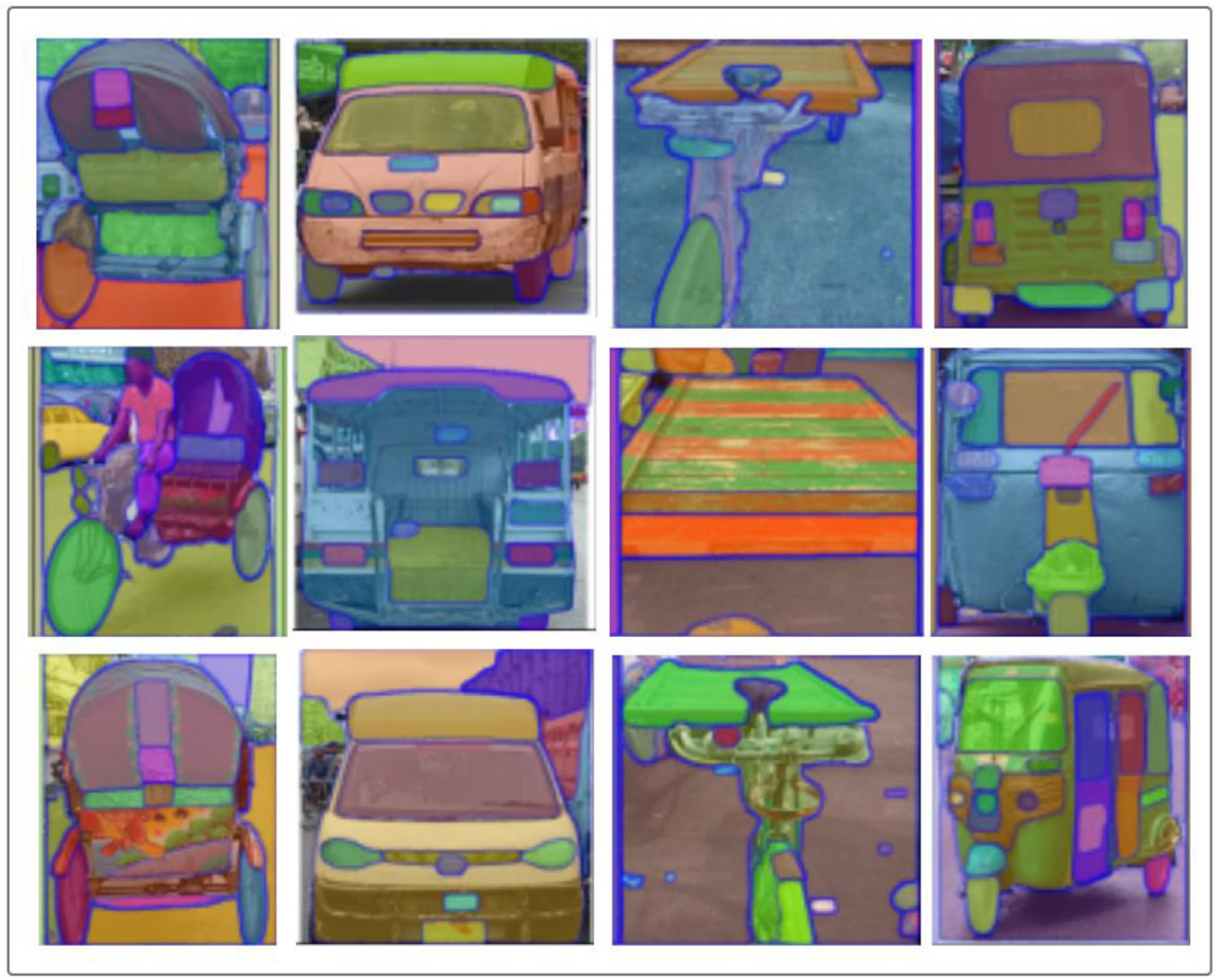
Illustrative semantic segmentation examples generated using the Segment Anything Model (SAM). These masks are provided for demonstration purposes only and are not included in the current dataset release.

Fig 3 illustrates semantic segmentation results for Auto Rickshaw images from the *AsianVehicle* dataset, generated using the Segment Anything Model (SAM) developed by Meta AI. SAM was applied in zero-shot mode, requiring no fine-tuning or manual annotation. These visualizations are provided for illustrative demonstration purposes only. The current dataset release does not include SAM segmentation masks, scripts, or configuration settings. Future dataset extensions may incorporate standardized annotation formats and segmentation masks to support object detection and segmentation benchmarking. Each colored region corresponds to an automatically detected segment, representing structural parts such as the windshield, door panels, wheels, and lights. The purpose of this visualization is to demonstrate how the dataset can support automated region identification and instance delineation for non-Western vehicles. The SAM framework enables flexible mask generation and interactive refinement, making it suitable for extending this dataset toward semi-supervised annotation, object-part analysis, and data-centric segmentation benchmarking. These examples highlight the dataset’s potential for exploring generalization and part-aware understanding across culturally specific vehicle forms.

## Limitations

While compiling the *AsianVehicle* dataset, several practical constraints were encountered. The images were collected exclusively from the Mirpur area of Dhaka, which may limit geographical diversity and representation of regional vehicle variations across Bangladesh. Future extensions will include additional regions and seasonal conditions. Data acquisition was performed only during daylight and within a moderate temperature range (25 °C–28 °C); hence, weather-related or seasonal variations are not reflected. No additional image enhancement or pre-processing was applied to maintain authenticity, which may result in inconsistent lighting or background conditions. Finally, occasional bystanders appearing near vehicles were anonymized through blurring, which may slightly alter localized visual detail in some frames.

## Ethics Statement

This dataset does not involve any studies with human participants or animals. All vehicle images were captured in public spaces using handheld devices, ensuring compliance with ethical data collection standards. To protect individual privacy, any identifiable human presence—such as faces or body parts visible near vehicles—was blurred or anonymized before inclusion. No license plates or personal identifiers are visible in the dataset. All images are original and collected directly by the authors without sourcing from social media or third-party archives. This data article does not reuse images from any previously published datasets.

## CRediT Author Statement

**Md. Darun Nayeem**: Investigation, Software, Validation, Formal Analysis, Methodology, Resources, Data Curation, Writing - Original Draft, Visualization. **Md. Sadikujjaman:** Data Curation. **Rehnuma Tabassum**: Data Curation. **Anika Ivnath:** Data Curation. **Md. Masudul Islam**: Project administration, Conceptualization, Methodology, Formal analysis, Resources, Writing - Original Draft, Visualization.

## Data Availability

Mendeley DataAsianVehicle: Image Dataset of Traditional Asian Vehicles for Computer Vision (Original data).
